# Optimization of Aflatoxin B1 Aptasensing

**DOI:** 10.1155/2017/2461354

**Published:** 2017-05-14

**Authors:** Marzieh Jafari, Mohsen Rezaei, Heibatullah Kalantari, Maryam Tabarzad, Bahram Daraei

**Affiliations:** ^1^Department of Pharmacology and Toxicology, School of Pharmacy, Ahvaz Jundishapur University of Medical Sciences, Ahvaz, Iran; ^2^Department of Toxicology, Faculty of Medical Sciences, Tarbiat Modares University, Tehran, Iran; ^3^Protein Technology Research Center, Shahid Beheshti University of Medical Sciences, Tehran, Iran

## Abstract

Combination of aptamers with DNAzymes attracted intense attention for development of DNA-based biosensors for detection of mycotoxins. In the present study a combination of aflatoxin B1 specific aptamer and HRP- (horseradish peroxidase-) mimicking DNAzyme was optimized for detecting aflatoxin B1. Detecting approach is based on the binding affinity of aflatoxin B1 to its specific aptamer and conversion of substrate to a detectable colorimetric signal by a linked DNAzyme. Compared to conventional methods for aflatoxin B1 detection, DNA-based assay has the advantages of low cost, long-term stability, and rapid, simple, and user-friendly steps.

## 1. Introduction

Mycotoxins are the well-known secondary metabolites of fungi with established adverse health effects in human and animals [1]. Aflatoxins are a group of mycotoxins produced by a variety of* Aspergillus* species including* A*.* flavus* and* A*.* parasiticus* [2]. Human contacts to these toxic metabolites are inevitable during preharvest, storage, or processing of agricultural products [3]. AFB1, the most toxic form of aflatoxins, has been linked to various health problems and has been placed in group 1 of human carcinogens by the International Agency for Research on Cancer (IARC) [4]. Food safety strategies and imminent contamination of crops with aflatoxins have increased the importance of AFB1 detection worldwide.

Various analytical methods have been developed for determination of AFB1 levels in food stuffs [5]. Despite their sensitivity and accuracy, chromatography based methods met some limitations that can be time consuming and expensive from sample preparation to the detection steps. Disadvantages of protein based assays are stability of antibodies under strict physical and chemical conditions and their long-term production processes. Consequently it is required to develop a rapid, simple, and low cost technique for AFB1 detection in food samples.

Numerous DNA sequences with enzymatic functions besides carrying of genetic information have been identified [6, 7]. Single-stranded DNAs because of their folding properties have been enrolled in many analytical procedures for developing of several DNA-based biosensors [8, 9].

The term aptamer refers to a single-stranded DNA which folds in to three-dimensional structure and interacts with its specific target [10]. This specific and short single-stranded oligonucleotide is chosen from a random sequence library and can bind to a wide variety of small molecule targets including proteins, nucleic acids, and cell receptors [11, 12]. DNAzyme, also known as deoxyribozyme, refers to a guanine-rich nucleic acid sequence with catalytic capabilities similar to common enzymes [13]. Several single-stranded DNA sequences with enzymatic activity in various colorimetric reactions have been identified [14].

DNAzymes and aptamers have remarkable advantages, including of low cost preparation and high efficiency [15, 16]. They are stable in different chemical or physical conditions. In contrast to proteins, they return to their original conformations when pH and temperature return to the initial condition [17, 18] and their combination has been recruited to analyze a wide range of molecules [19–22]. Based on these properties, many DNA-based biosensors have been introduced for detection of mycotoxins [23, 24].

**Scheme 1:**
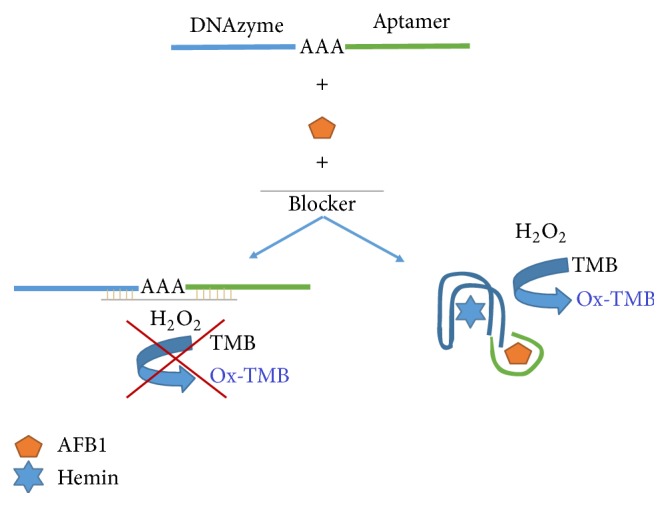
Schematic representation of AFB1 detection by conjugated DNAzyme-aptamer.

In the present study a combination of AFB1 specific aptamer [25] and HRP- (horseradish peroxidase-) mimicking DNAzyme [13] was optimized for detecting of AFB1 (Scheme 1). Binding of AFB1 to its specific aptamer recognition sequence prevents the blocker from being annealed to the aptamer and the reaction proceeds to yield a blue color product in a concentration dependent manner.

## 2. Materials and Methods

### 2.1. Reagents

Aflatoxin B1 (AFB1), Tris HCl, sodium chloride (NaCl), magnesium chloride (MgCl2), 3,3,5,5-tetramethylbenzidine (TMB) are purchased from Sigma (USA). Hemin was purchased from Serva (USA). All the chemical reagents were of highest grade and used without further purification. All solutions were prepared with diethyl pyrocarbonate (DEPC) treated deionized water.

Oligonucleotides contained a sequence of 49 bp aflatoxin B1 aptamer [25] and 18 bp DNAzyme [13] in two different sequential orders (5′-aptamer-DNAzyme-3′ or 5′-DNAzyme-aptamer-3′) and blockers complementary sequences were purchased from Biolegio (Netherlands). These sequences are shown in Table 1. Mfold software was used for prediction of the secondary structure of used single-stranded nucleic acids [26].

### 2.2. Assay Procedure

**Scheme 2:**
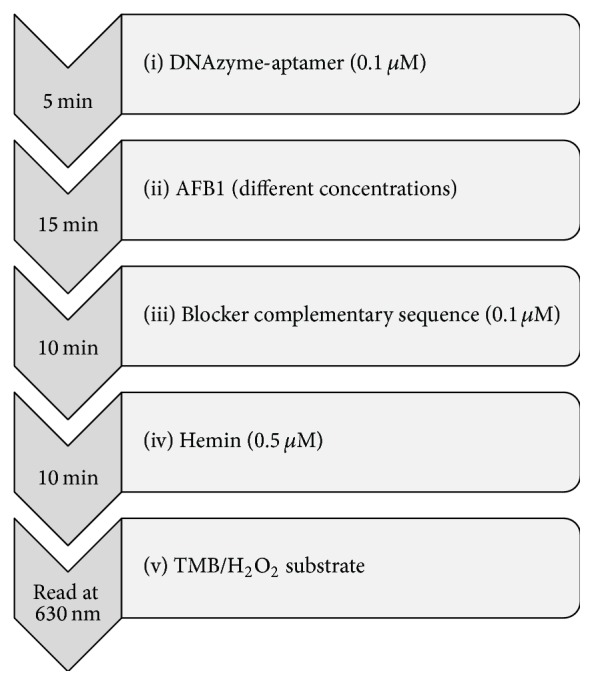
Schematic representation for optimized assay procedure.

DNA stock solutions (100 *μ*M) were prepared in DEPC deionized water and stored in small aliquots. DNA working solutions were prepared in incubation buffer (10 mM Tris–HCl, pH 8, 120 mM NaCl, 2.5 mM MgCl2, and 5 mM KCl) [22]. Before starting the experiments oligonucleotides were denatured at 95°C for 5 min. Then, 70 *μ*L of AFB1 aptamer (final concentration of 0.1 *μ*M) incubated with different concentrations of 10 *μ*L of AFB1 for 15 min. 10 *μ*L of 1 *μ*M blocker complementary sequence was then added in to 96-well microplate and incubated for 10 min at room temperature. 10 *μ*L of 0.5 *μ*M hemin was also added to wells followed by 10 min incubation. TMB substrate containing H_2_O_2_ was prepared immediately before use and 100 *μ*L of substrate solution was added to the mixture. For kinetic assay, absorbance of TMB color product was measured at wavelength of 630 nm for 5 min (Scheme 2). All absorbances were measured by a BioTek (ELx800) microtiter plate reader (BioTek, USA).

## 3. Results and Discussion

**Figure 1:**
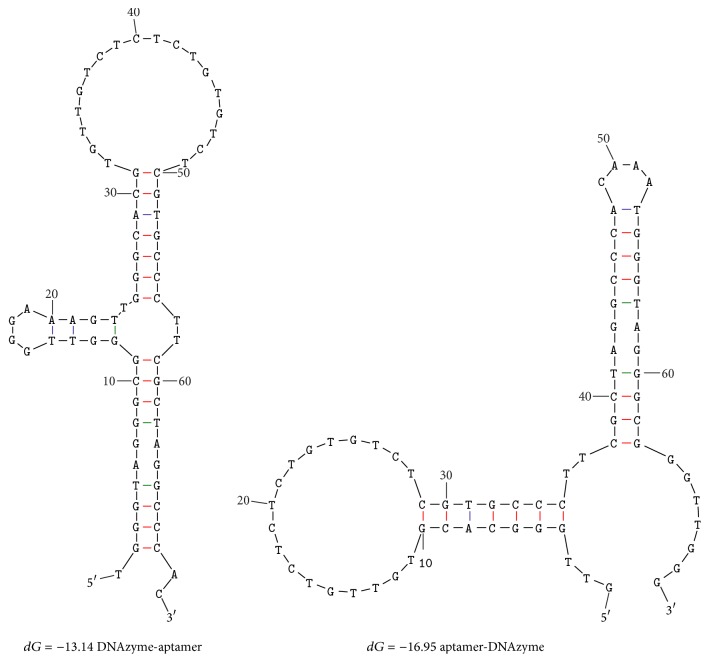
Predicted secondary structure of DNAzyme-aptamer and aptamer-DNAzyme using Mfold tool.

DNAzyme sequence can attach to either 5′ or 3′ ends of aptamer sequence yield DNAzyme-aptamer or aptamer-DNAzyme (Table 1 and Figure 1). In our previous study, we investigated the relationship between the orientation of the DNAzyme and aptamer conjugation and their final peroxidase activities [27]. As seen in Table 2, DNAzyme-aptamer displayed a higher enzymatic activity than 3′ oriented conjugation. Aptamer-DNAzyme revealed its priority for further evaluations in biosensor design.

**Figure 2:**
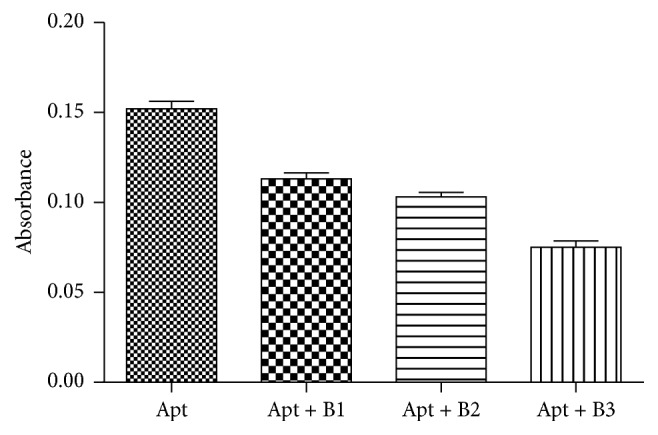
Blockade of peroxidase activity of DNAzyme-aptamer by B1, B2, and B3 sequences. Apt: DNAzyme-aptamer; B: blocker complementary sequence.

For achieving of best results, several parameters were optimized including blocker complementary sequences (B1, B2, and B3) selection, incubation times, reagent concentrations, and their order of addition. Optimal annealing to DNAzyme-aptamer and inhibiting of its enzymatic activity were influenced mainly through the blocker complementary sequence. B1, B2, and B3 were designed with different numbers and sequences of nucleotides. As shown in Figure 2 the blockade of peroxidase activity has considerably been attained by B3 sequence compared to B1 and B2.

**Figure 3:**
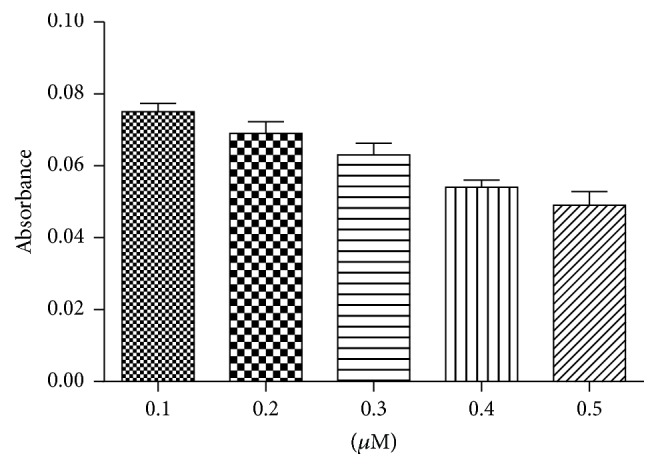
Blockade of peroxidase activity of DNAzyme-aptamer by increasing concentration of B3.

At the next step, different molar ratios of DNAzyme-aptamer and B3 blocker from 1 : 1 to 1 : 5 were tested. With a constant amount of DNAzyme-aptamer (0.1 *μ*M), increasing the blocker concentration from 0.1 to 0.5 *μ*M resulted in a concentration dependent decline of DNAzyme activity (Figure 3). Given that AFB1 and complementary sequence of blocker compete with each other for binding to DNAzyme-aptamer, blocker at high concentration decreases the sensitivity of assay. So a conservative lower molar ratio of 1 : 1 was selected for further evaluations.

Orders of addition of AFB1, blocker complementary sequence, and hemin were evaluated in different settings. Results showed that adding blocker prior to AFB1 resulted in unaffected remaining of blocker at its binding site. Therefore AFB1 was scheduled to incubate with aptamer before the blocker addition. Also we observed that enzymatic activity will be temporally influenced when hemin is added to the reaction containing blocker. As hemin prevented the hybridization of blocker complementary sequence and DNAzyme-aptamer, it was added subsequently to the blocker sequences addition.

The best incubation times for AFB1, blocker complementary sequence, and hemin were achieved via incubation of all reagents separately at 10 min intervals up to 60 minutes. The shortest and most efficient incubation times were 15, 10, and 10 min for AFB1, blocker, and hemin, respectively.

**Figure 4:**
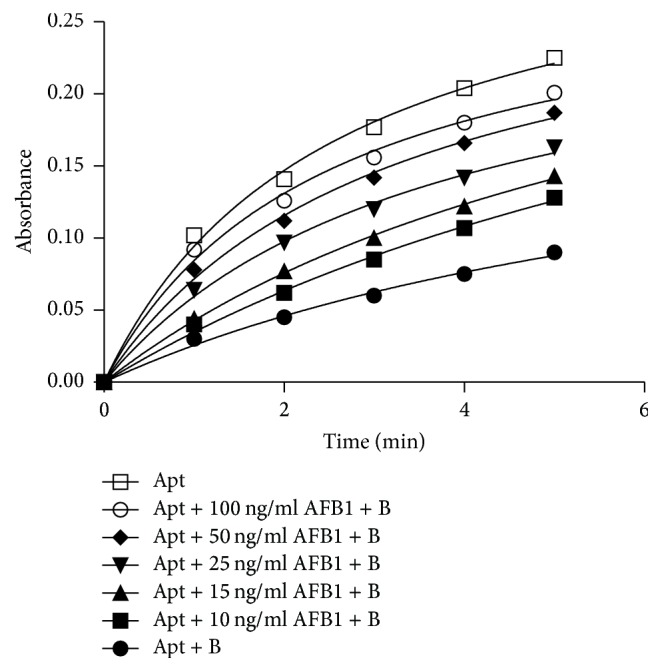
Peroxidase activity of DNAzyme-aptamer in the presence of B3 and different concentrations of AFB1. Apt: DNAzyme-aptamer, B: blocker complementary sequence, and AFB1: aflatoxin B1.

In the present work, the sensing strategy is based on the binding affinities of AFB1 to its specific aptamer that produce a detectable colorimetric signal by DNAzyme (Scheme 1). In the absence of AFB1, annealing of blocker sequence (complementary sequence to a part of DNAzyme-aptamer) to DNAzyme-aptamer decreases the enzymatic activity. In the presence of AFB1, the aptamer binds to AFB1 and forms a hairpin structure. Consequently, blocker complementary sequence was prevented from being bound to DNAzyme-aptamer and following addition of hemin, DNAzyme displays a colorimetric signal that is directly associated with AFB1 concentrations (Figure 4).

Under optimal conditions the limit of detection of 10 ng/mL was achieved. AFB1 aptamer has been used as a recognition probe in several detection systems based on PCR, electrochemical, chemiluminescent, colorimetric, and fluorescent platforms. In Guo et al.'s study, AFB1 aptamer with 3′-terminal biotin groups has been immobilized on the surface of PCR tubes for developing of an aptasensor (LOD: 25 fg/mL) based on RT-qPCR [28]. An aptamer-based dipstick assay (LOD: 0.1 ng/mL) using biotin-modified aptamer has also been reported by Shim et al. [29]. In another work by Shim et al. based on chemiluminescence competitive assay, AFB1-OVA conjugate was coated on the wells (LOD: 0.11 ng/mL) [30]. Castillo et al. have developed an aptamer-based biosensor (LOD: 0.40 nM) using immobilization of amino-modified aptamers and electrochemistry [25].

One of the important goals of this work was designing a simple and cost-effective method for AB1 detection, without intricate steps and equipment. In the mentioned publications, aptamer has been modified with functional groups or immobilized on surfaces. In our experiment, AB1 aptamer has been employed without any immobilization and modification. All steps of procedure were done as a simple solution. Since aptamer interaction with its target depends on folding into unique structures [31], intact aptamers were excepted to have more appropriate folding.

AFB1 aptamer and DNAzyme also have also been used by Seok et al. with some alterations [32]. In their study, two split DNAzyme halves anneal with aptamer that form G-quadruplex. The AFB1 aptamer complex prevented the annealing of split DNAzyme and aptamer, therefore weak color intensity will be observed upon addition of ABTS substrate. The accuracy of this method is depending on the correct annealing of two split DNAzymes with aptamer. The amount of this annealing may be variable in each performance and cause false negative results. In our work, to have the stable signal, DNAzyme sequence has been attached to 5′ ends of aptamer sequence. Therefor its catalytic activity remained constant in all experiments. Also in their study all reagents including split DNAzyme probes, aptamer, hemin, and AB1 were added simultaneously, while our results showed that the order of addition of reagents is an important parameter.

## 4. Conclusions

Advantages of using DNAzymes and aptamers over protein enzymes and antibodies have been reported in many studies. In this study, we optimized a colorimetric simple assay using DNAzyme-aptamer conjugate to detect AFB1. Under optimized conditions, the formation of AFB1 aptamer complex prevents the hybridization of its complementary sequences. Hence, the catalytic activity of DNAzyme increases corresponding to AFB1 concentration. To improve the procedure, we will work on the limit of detection and sensitivity of this aptasensor for a more accurate and sensitive determination of AFB1.

## Figures and Tables

**Table 1:** Sequences of oligonucleotides used in this study. ssDNA: single stranded DNA; B: blocker complementary sequence.

ssDNA	Sequences (5′-3′)
DNAzyme-aptamer	TGGGTAGGGCGGGTTGGGAAAGTTGGGCACGTGTTGTCTCTCTGTGTCTCGTGCCCTTCGCTAGGCCCAC
Aptamer-DNAzyme	GTTGGGCACGTGTTGTCTCTCTGTGTCTCGTGCCCTTCGCTAGGCCCACAAATGGGTAGGGCGGGTTGGG
B1	CACGTGCCCAACAAATCCCAACCC
B2	CTGACAGAGAGAAACCACGTGCCCAACAAATCCCAACCC
B3	GAGAGACAACACGTGCCCAACAAATCCCAACCCGCC

**Table 2:** Kinetic parameters for DNAzyme-aptamer and aptamer-DNAzyme catalytic activity.

	DNAzyme-aptamer	Aptamer-DNAzyme
*V*_max_ (mM/s)	0.06	0.02
*K*_*m*_ (mM)	0.6	0.4
